# MLgsc: A Maximum-Likelihood General Sequence Classifier

**DOI:** 10.1371/journal.pone.0129384

**Published:** 2015-07-06

**Authors:** Thomas Junier, Vincent Hervé, Tina Wunderlin, Pilar Junier

**Affiliations:** 1 Laboratory of Microbiology, University of Neuchâtel, Neuchâtel, Neuchâtel, Switzerland; 2 Vital-IT Group, Swiss Institute of Bioinformatics, Lausanne, Vaud, Switzerland; 3 Laboratory of Biogeosciences, Institute of Earth Sciences, University of Lausanne, Lausanne, Vaud, Switzerland; Georgia Institute of Technology, UNITED STATES

## Abstract

We present software package for classifying protein or nucleotide sequences to user-specified sets of reference sequences. The software trains a model using a multiple sequence alignment and a phylogenetic tree, both supplied by the user. The latter is used to guide model construction and as a decision tree to speed up the classification process. The software was evaluated on all the 16S rRNA gene sequences of the reference dataset found in the GreenGenes database. On this dataset, the software was shown to achieve an error rate of around 1% at genus level. Examples of applications based on the nitrogenase subunit NifH gene and a protein-coding gene found in endospore-forming Firmicutes is also presented. The programs in the package have a simple, straightforward command-line interface for the Unix shell, and are free and open-source. The package has minimal dependencies and thus can be easily integrated in command-line based classification pipelines.

## Introduction

Reconstructing environmental communities of microorganisms often involves identifying lineages from a nucleotide or protein sequence. The identification will typically be sought at some predetermined taxonomic rank (for example species or genus). Classification then consists of assigning a given sequence, called the *query sequence*, to a single taxon, among many of the chosen rank. The set of taxa among which the query is to be classified are termed the *reference taxa*.

According to a recent study by Bazinet and Cummings [1], classification methods fall into one (rarely two) of the three following categories: similarity-, composition-, and phylogeny-based. Similarity methods compare the query to a set of references using sequence-level similarity measures (for example with BLAST [2] or hidden Markov models), and classify according to which OTU(s) is most similar to the query. Examples of pipelines that implement this classification approach include MEGAN [3], CARMA3 [4] and MG-RAST [5]. Composition methods use machine-learning techniques to classify queries based on properties of the sequences, such as frequencies of fixed-length words (k-mers). The Ribosomal Database Project (RDP) classifier [6] as well as SCIMM [7] and TACOA [8] belong to this category. Finally, phylogeny-based methods classify by placing the query in a phylogenetic tree along with references and examining its relatives. To this class belong, among others, EPA [9] and pplacer [10].

Still according to Bazinet and Cummings [1], there are caveats to each approach. Similarity methods were found to be generally accurate, but may be slow when the reference database is large. Composition methods incur an overhead for building the model, but then are typically faster than similarity methods. Phylogenetic methods are able to assign queries not only to the leaves of the tree but to higher ranks as well, although they tend to use CPU-intensive methods.

Besides the scope and classifying method, the criteria that a regular user might consider while selecting any of the aforementioned classification tools include the type of sequencing project (amplicon versus shotgun sequencing) or the targeted gene(s). In addition, criteria like interface (graphical or command-line, local or web-based) may guide the selection of a software tool.

In our research projects, we needed to classify relatively short (200 amino acids or less) protein sequences from essentially unknown environmental samples. The choices for classification are thus restricted to a few options. One alternative is to use a similarity-based method such as BLAST on a customized database of references. Another is to train a gene-specific classifier on the gene of interest. The latter is the case of the RDP, which is commonly used for classifying 16S rRNA genes. However, for the RDP, classification based on amino acid sequences was not possible when tried in our project, which is an issue for protein-coding genes. We therefore designed and implemented a classification program, MLgsc, with the following properties:

- it classifies both protein and nucleic acid sequences;
- it is intended for targeted classification (e.g. amplicon sequencing);
- it builds a model using a multiple sequence alignment of reference sequences from the classifying region, and a phylogenetic tree of the references;
- it uses position-specific weight matrices (PWM) to measure a query’s similarity to a set of references (therefore, it falls in Bazinet and Cummings’ “similarity” category);
- it uses a phylogeny to avoid comparing the query to all models of individual references (and therefore arguably belongs in the “phylogeny” category as well);
- it has minimal dependencies, in the sense that it does not call other analysis programs or functions;
- it has a simple interface: training the model and using it to classify sequences are each performed as a single shell command involving at most a few arguments and options. This makes it straightforward to include in shell-based classification (or other) pipelines.

## Interface

The MLgsc package consists of three programs: mlgsc_xval, mlgsc_train, and mlgsc, which perform cross-validation, training, and classifying, respectively (see Table 1).

**Table 1:** The MLgsc programs and their functions, inputs, and outputs.

Program	Function	Inputs	Outputs
mlgsc_xval	cross-validation	multiple alignment^(a)^, phylogeny ^(b)^	predictions^(c)^
mlgsc_train	training classifier^(d)^	multiple alignment^(a)^, phylogeny^(b)^	classifier^(e)^
Mlgsc	classification^(d)^	query sequences^(f)^, classifier^(g)^	predictions^(h)^

(a) A multiple alignment provided by the user of known sequences from the gene or protein of interest, in Fasta format.

(b) A phylogeny of the reference taxa provided by the user in Newick format.

(c) A text output, containing (among others) the actual and predicted taxon names, thus enabling the detection of classification errors.

(d) This step is conditioned on a successful cross-validation (mlgsc_xval).

(e) This is a binary file, to save space and to save reading and parsing time

(f) A Fasta file containing the sequences to be classified, usually unaligned.

(g) The output of mlgsc_train.

(h) A text file that contains the predicted assignment to reference taxa for each query sequence (among other information).

## Procedure

Classifying using MLgsc involves the following steps:

1. selecting the scope, classifying region, and molecule type
2. obtaining a multiple alignment of reference sequences and a phylogeny of the reference taxa.
3. validating the alignment and tree for classification
4. building a classifier
5. using the classifier

### Scope and Classifying Region

The first step is the selection of the scope of the classifier, that is, the set of taxa the classifier will recognize from the query sequences. This can be a relatively small clade like the Firmicutes at species level, or it can be more encompassing, such as all prokaryotes at genus level. One then selects a classifying region—a conserved region that will serve as the basis for classification. This can be dictated by experiment design (for example a region targeted by PCR); in any case the region should be well-enough conserved in all reference taxa so as to yield good-quality multiple sequence alignments, but should include sufficient variation to distinguish between them. For protein-coding regions, one has the option of classifying at the nucleotide or the amino acid level – an MLgsc classifier can classify either type of sequence, but not a combination of both.

### Multiple Alignment and Phylogeny

One then obtains a multiple alignment of reference sequences of the classifying region. This can be directly downloaded from sites like GreenGenes [11], or it can be computed from reference sequences using any suitable program such as Muscle [12] or MAFFT [13]. The sequences should cover the entire classifying region. Each taxon should be represented by at least one reference sequence. Experiments show that providing more than a dozen sequences per reference taxon does not increase accuracy, at least at genus level for 16S rRNA. The alignment should be in gapped FASTA format. The FASTA header should have an ID followed by the taxon name, separated by white space, for example

>AAJGZX Clostridium

ACTGCTG—-GTA…

>AB67CH Butyrivibrio

ACTTGCC—-GCA. …

In this example, the taxon names are *Clostridium* and *Butyrivibrio*. The IDs are not part of the model, but they are useful in the validation step (see below), for identifying any problematic sequences.

Finally, one needs a phylogenetic tree of the references, in Newick format [14]. This can be computed directly from the alignment using any tree-building software such as PhyML [15] or RaxML [16]. Alternatively it can be derived or extracted from external sources, for example NCBI’s taxonomy [17] or the All-species Living Tree [18]. The tree’s leaf labels should be the same reference names as in the alignment. Below is an example tree in Newick format:

((Bacillus,Paenibacillus)Bacillaceae,(Clostridium,(Butyrivibrio,Marvinbryantia)Lachnospiraceae)Clostridiales)Firmicutes;

Inner node labels, that is, names of clades above the level of the reference taxon (e.g. Bacillaceae in the above example) are allowed and in fact recommended, as they allow a more detailed output. MLgsc deliberately does not include functionalities to compute multiple alignments or phylogenetic trees. This gives the user complete freedom in selecting state-of-the-art sources or tools to perform these steps prior to classification.

### Validation

To verify that a classifier based on the data obtained above will be accurate enough, the user can perform a cross-validation. The program mlgsc_xval (“mlgsc cross-validate”) performs one particular form of cross-validation called *leave-one-out*: one sequence is drawn at random, without replacement (i.e., is left out), from the input alignment, and the classifier is built with all the remaining sequences. The single, left-out sequence is then used as a query, and the prediction is considered correct if the sequence's predicted taxon matches its actual one. Taxa represented by only one reference sequence cannot be used in this way, since the query taxon must also be represented in the classifier. By default, only taxa with three or more reference sequences are considered suitable.

The procedure is repeated a number of times, (by default, 100, or every sequence if there are less than 100 suitable sequences), and the number of errors divided by the number of trials is an estimate of the error rate of the classifier.

Cross-validation is performed by a command like the following:

$ mlgsc_xval Prot alignment.msa phylogeny.nw

in which Prot specifies that the classifier is for proteins, and alignment.msa and phylogeny.nw are the names of the alignment and phylogenetic tree files obtained in the previous step, respectively. This command produces output like the following:

ZQBXK1 Bacillus-> Bacilli (71); Bacillaceae (101); Bacillus (83)

AAJGZX Clostridium-> Clostridia (116); Clostridiales (163); Clostridium (153)

AB56K Dorea-> Clostridia (132); Clostridiales (21); Clostridium (9)

The number in parentheses after the node name is a confidence measure. It is the logarithm base 10 of the evidence ratio of the best-scoring position-specific weight matrix (PWM) to the next-best, i.e. the ratio of their likelihoods given the aligned query sequence. Mis-classified sequences often have a low confidence measure for at least one node in their predicted classification (such as the value of 9 above for *Clostridium*) In this example, *Dorea* is very confidently (and correctly) classified in the Clostridia, less confidently (but still correctly) in the Clostridiales, and not confidently (and wrongly) in *Clostridium*.

The confidence measure can be used to detect sequences for which MLgsc cannot find a confident prediction. This is similar to the bootstrap-based confidence values provided by RDP [6].

In the example above, each line corresponds to a leave-one-out trial, with a new sequence drawn at random and a new classifier trained from all the others. The last line shows a misclassification (*Dorea* wrongly classified as *Clostridium*). mlgsc_xval has an option (-x) that causes it to print only wrong classifications.

### Training

Once the user is satisfied that the classifier is sufficiently accurate, a classifier is trained and saved in a file. This is done as in the following command:

$ mlgsc_train–o taxon.mod Prot alignment.msa phylogeny.nw

where–o taxon.mod specifies the name of the output file, Prot specifies that the sequences are proteins, alignment.msa is the multiple alignment and phylogeny.nw is the phylogenetic tree. The output file is the classifier itself.

### Classification

Finally, the classifier built in the previous step can be used to classify query sequences as in the following example:

$ mlgsc queries.fasta taxon.mod

where queries.fasta is a FASTA file containing the sequences to classify (there are no particular requirements other than that they be in FASTA format) and taxon.mod is the model file, produced as shown above. The output is a TAB-separated plain text file with query ID, predicted reference, path and confidence measure at each level in the decision tree:

A0RIG4_BACAH-> Bacilli (71); Bacillaceae (101); Bacillus (83)

A0Q0B0_CLONN-> Clostridia (116); Clostridiales (163); Clostridium (153)

A5Z6L5_9FIRM-> Clostridia (132); Clostridiales (*); Clostridium (9)

…

In the example above we see that A0RIG4_BACAH is classified as a *Bacillus*, while A0Q0B0_CLONN and A5Z6L5_9FIRM are classified as *Clostridium*, the latter with lower confidence regarding the genus (confidence measure 9 versus 153). An unlabeled node in the tree appears as unnamed in the classifier output. When an evidence ratio is very large (logarithm greater than 1000), it is represented by an asterisk (*).

## Algorithm

### Training the Model

**Fig 1:**
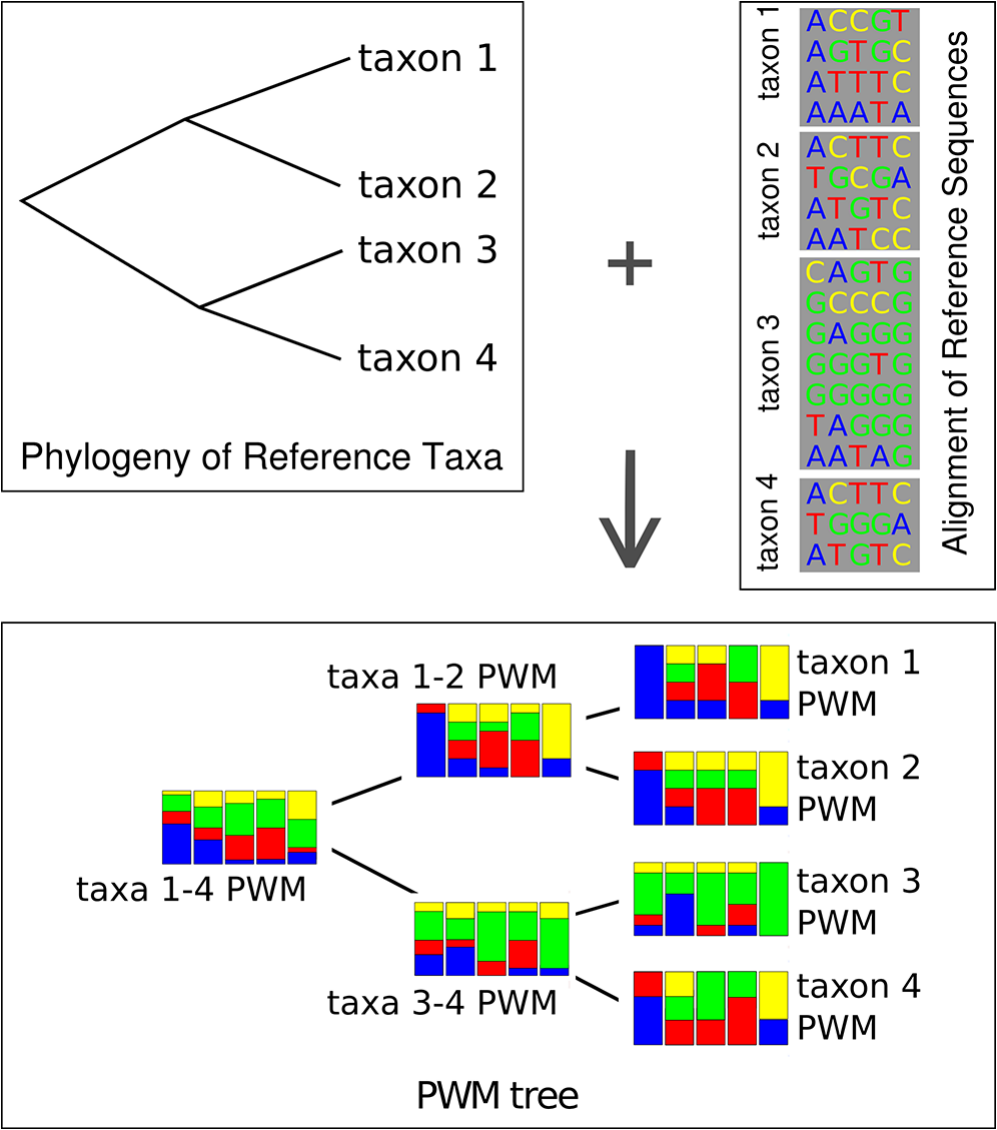
Training the Model. A tree of position-specific weight matrices (bottom) constructed from a phylogeny (top left) and a multiple alignment (top right). Each taxon in the tree is represented by at least one (preferably more) sequence(s) in the alignment. Column *i* in a taxon matrix contains the relative frequencies of residues at position *i*, computed over all sequences of that taxon. Matrices at inner nodes are averages of the matrices of the node’s children. The tree need not be bifurcating, but a fully bifurcating tree offers the best speed performance (see Discussion).

MLgsc constructs a tree of position-specific weight matrices (PWMs) using a multiple alignment of sequences from the classifying region and a phylogenetic tree of the reference taxa (Fig 1). The tree of PWMs has the same topology as the phylogeny of the taxa, and a matrix in the PWM tree models a clade in the phylogeny: a matrix at a tip of the tree models a single taxon, while a matrix in an internal tree node models a clade of two or more taxa. Column *i* in a matrix contains the relative frequencies of residues at position *i*, computed over all reference sequences belonging to the clade modeled by the matrix. It therefore represents a maximum-likelihood estimate of the true frequencies in the wild. Gaps in the alignment are simply considered an additional character, so the matrix models the probability of occurrence of a deletion with respect to the alignment.

### Probability and Score of a Sequence given a Matrix

Given a matrix *M* of length l, denoting f_i_(r) the frequency of residue r at position i in *M*, and under the assumption of independence of the columns of M, the probability of a sequence r_1_r_2_…r_l_ given M is $p=\prod_{i=1}^{l}f_{i}\left(r_{i}\right)$. It is impractical to use probabilities directly, because they tend to be so close to zero as to cause underflow. A common solution to this problem is to use logarithms of probabilities: this avoids numbers very close to zero, and also replaces multiplications by sums, which are usually faster. MLgsc also rounds the logarithms, which enables it to use integer arithmetic, which is faster than its floating-point counterpart. The value thus computed is called the *score* of a sequence given a matrix: $score=\sum_{i=1}^{l}\left\lfloor k\;\text{log}\left(f_{i}\left(r_{i}\right)\right)\right\rfloor$ (where *k* is a scaling constant). A PWM assigns a *score* to any sequence of the same length as itself. In our case, the score is an estimate of the probability *P(s|M)* of the sequence given the matrix, under the assumption that the probabilities of any two residues in the sequence are independent – an assumption that is false in general but useful in practice. Naive Bayesian classifiers such as RDP [6] make a similar assumption, from which their name is derived.

Considered as a function of *M* and keeping the query sequence *s* fixed, *P(s|M)* is the *likelihood* of a matrix, and MLgsc reports the reference whose associated matrix maximizes this function. This report includes the entire path through the tree, including confidence measures at each level (see example of output below).

#### Bias reduction

To reduce biases due to over-sampling of the same sequence in an OTU, and over-representation of some OTUs with respect to others, the following step is taken:

Before constructing the PWMs, the aligned sequences are weighted according to Henikoff and Henikoff [19]: the Henikoff weight of each sequence is computed, each weight is divided by the smallest of the weights and rounded up to the nearest integer, yielding an adjusted weight. Each sequence is then repeated as necessary so that its frequency in the weighted alignment equals its adjusted weight. No sequence has an adjusted weight smaller than one.

This weighting is performed only on the whole input alignment. We experimented with weighting every clade separately, but found that it had little effect.

### Classifying Queries

**Fig 2:**
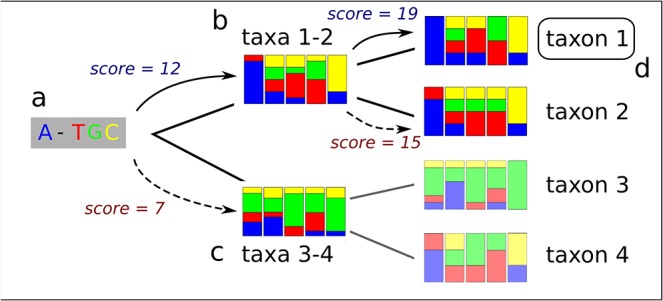
Classifying. The aligned query sequence (a) is first scored by the matrices at the root’s direct children nodes, in this example the matrix for taxa 1 and 2 (b) and the one for taxa 3 and 4 (c). Matrix (b) is found to yield the better score (solid arrow). Therefore, the query is now scored against matrix (b)’s children, namely taxa 1 and 2. The former is found to yield the better score, and OTU 1 is reported as the most likely (d). The shaded parts of the tree (matrices for taxa 3 and 4) are never tested. In a balanced, fully bifurcating tree of n nodes, only 2log_2_(n) matrices are tested.

The query sequence is first aligned to the PWM at the root of the tree, using semi-global alignment (local in the query and global in the PWM). The method uses dynamic programming similar to Needleman and Wunsch’s algorithm [20], but with match/mismatch scores based on weights in the PWM instead of being fixed, and gaps disallowed in the PWM. The aligned query is thus exactly as long as the PWM. The aligned sequence is then scored against the matrices at the children nodes of the root. The matrix yielding the highest score is selected, and the sequence is now scored against the children of this matrix, and so on recursively until the highest-scoring matrix is at a leaf (Fig 2). The corresponding reference is reported as the most likely, together with the path through the tree. At each node, the logarithm base 10 of the evidence ratio between the best-scoring and next-best scoring PWM is shown, which serves as a measure of confidence in the decision taken regarding classification at each step. In this case, the evidence ratio is simply the ratio of likelihoods between two PWMs.

## Performance and Comparisons

Using leave-one-out cross-validation with mlgsc_xval on a set of 16S rRNA gene sequences obtained from the GreenGenes database release 13 (11954 sequences of 585 bacterial genera) and a bacterial phylogeny downloaded from NCBI, the error rate of MLgsc was estimated at around 1%.

We compared MLgsc's speed and accuracy with other classifiers, as summarized in the tables below. All tests but one were carried out on a single core of a 2.30 GHz Intel Core i7-4712MQ with 16 GB RAM running Ubuntu Linux 14.04. The exception was suffix tree search, which was carried out on 2 Intel Xeon E5-2260 (16 cores, 2.20 GHz) and 128 GB RAM running Scientific Linux release 6.

### 16S rRNA genes

The test set consisted of the same 11,954 16S rRNA sequences from GreenGenes 13 (See Accuracy, above) MLgsc was compared with several classifying methods implemented in Mothur version 1.34.4 [21] using GreenGenes for reference (Table 2). Mlgsc was itself trained on GreenGenes v. 13, using a phylogeny derived from NCBI Taxonomy.

**Table 2:** Comparison of speed and accuracy of MLgsc versus classifying methods implemented in Mothur for the 16S rRNA gene.

Method	Run Time [s]	Classified ^(a)^	% Classified	Wrong	Error rate [%]
Mothur (RDP)^(b)^	5,590	11,935	99.84	382	3.2
Mothur (KNN, k-mer)^(c)^	396	8,165	68.3	0	0
Mothur (KNN, BLASTN)^(d)^	17,162	8,432	70.54	4	0.047
Mothur (KNN, suffix tree)^(e)^	3,543	7,995	66.88	0	0
Mlgsc (no ER cutoff)^(^ ^a^ ^,^ ^f^ ^)^	35	11,954	100	297	2.5
MLgsc (ER cutoff 10)^(^ ^a^ ^,^ ^f^ ^)^	35	11,247	94.09	65	0.58
MLgsc (ER cutoff 20) ^(^ ^a^ ^,^ ^f^ ^)^	35	10,041	84	15	0.15

(a) For the Mothur methods, a query was considered not classifiable if the corresponding output line did not indicate a genus (g__ prefix). For MLgsc, a query was considered not classifiable if any node in the corresponding output line had an evidence ratio (ER) below the cutoff. The use of evidence ratio do detect sequences that are not confidently classified is described in section Procedure, subsection Validation.

(b) Mothur command: classify.seqs(fasta = aln, template = gg_13_8_99.fasta, taxonomy = gg_13_8_99.gg.tax, iters = 1000, method = wang, ksize = 8, processors = 1)

(c) Mothur command: classify.seqs(fasta = aln1, template = gg_13_8_99.fasta, taxonomy = gg_13_8_99.gg.tax, iters = 1000, method = knn, numwanted = 10, search = kmer, ksize = 8, processors = 1)

(d) Mothur command: classify.seqs(fasta = aln2, template = gg_13_8_99.fasta, taxonomy = gg_13_8_99.gg.tax, iters = 1000, method = knn, numwanted = 10, search = blast, processors = 1)

(e) Mothur command: classify.seqs(fasta = aln3, template = gg_13_8_99.fasta, taxonomy = gg_13_8_99.gg.tax, iters = 1000, method = knn, numwanted = 10, search = suffix, processors = 16)

(f) MLgsc command: mlgsc-A 16S.fasta 16S_classifier.bcls

In all cases the query sequences were aligned prior to classification (i.e. the input to the classifiers was a multiple alignment) because this is required by Mothur.

### NifH

As an example of classification at the protein level, we chose the nitrogenase iron protein 1 (NifH). This protein is involved in nitrogen fixation and is found in a wide variety of Archaea and Bacteria. MLgsc was compared with the FunGene Pipeline's FrameBot component [22], which performs protein-coding gene classification (Table 3).

**Table 3:** Comparison of speed and accuracy of MLgsc versus FrameBot for the nitrogenase gene *nifH*.

Method	Run Time [s]	Classified ^(a)^	% Classified	Wrong^(b)^	Error rate [%]
FrameBot	100.87	422	100	103	24.41
MLgsc (no ER cutoff)	4.9	383	100	52	13.58
MLgsc (ER cutoff 10)	4.9	318	83.03	8	2.52

(a) For MLgsc, a query was considered not classifiable if any node in the corresponding output line had an evidence ratio (ER) below the cutoff. The use of evidence ratio do detect sequences that are not confidently classified is described in section Procedure, subsection Validation.

(b) A FrameBot classification was evaluated by examining the lines starting by STATS in the *framebot.txt output file:, which contains the predicted genus name and the test sequence's ID, for example in STATS 454423|B|1|1594_2487_L23514 **Nostoc**_commune_UTEX_584_nitrogen_fixation_protein_nifU**AAA21838** the predicted genus is *Nostoc* and the query ID is AAA21838. Using the ID to look up the genus in the reference file, we consider the prediction correct if the two genera match.

(c) FrameBot command: java-jar dist/FrameBot.jar framebot-N-o test_nifH refset/nifh_prot_ref.fasta ENA_nifH_full_cleanHdr.dna

(d) MLgsc command: mlgsc ENA_nifH_full_cleanHdr_orf800_bestORF.pep NifH_ref_clean_train.bcls

We downloaded all sequences from the European Nucleotide Archive (ENA) [23] that met the following criteria: “Coding” section, prokaryote (PRO) division, “nifH” gene name, and sequence length no greater than 1000 bp. This yielded 5,825 sequences. We further excluded all sequences whose description contained the word 'partial', yielding 444 sequences with a median length of 882 bp. The nucleotide sequences were (a) classified directly by FrameBot; and (b) translated then classified with MLgsc. ORFs shorter than 800 bp were discarded, and when a sequence yielded more than one ORF of sufficient size, only the best one was classified. The best ORF was defined as the one yielding the highest score when compared to the reference NifH protein sequences by global pairwise alignment with EMBOSS's needleall program. The final set of translated ORFs contained 383 sequences.

## Example of an Application

MLgsc was also tested on a set of amplicon sequences obtained for the *spo0A* gene. This molecular marker has been demonstrated to be specific to endospore-forming bacteria [24] and was used to study the diversity of this bacterial group in environmental samples [25]. A sediment sample from Lake Geneva (46° 27.03 N, 6° 42.52 E, at 284 m depth) was collected during a research campaign with the MIR manned submersibles in June 2011. A 602-bp sequence of the *spo0A* gene was amplified as previously described [26]. PCR reactions were done in quintuplets that were pooled and purified with a MultiScreen PCRμ96 plate (Merck Millipore) and afterwards eluted in 20 μl molecular grade sterile water. The purified samples were loaded onto a 1% agarose gel and electrophoresis run for 40 min at 80 V. The bands of the correct size (602 bp) were excised and purified with a QiaQuick Gel extraction kit (QIAGEN). Purified amplicons were then sent to Eurofins MWG Operon for barcode amplicon sequencing with Roche GS FLX+. A set of 1,174 sequence reads was obtained, of which 81% was retained after quality control.

For quality control, the nucleotide sequences were translated to their amino acid sequences, using the EMBOSS [27] package’s transeq utility. The amino acid sequences were then aligned and compared to a Gribskov-style protein profile [28] of Spo0A sequences that was built based on 27 known Spo0A sequences as described elsewhere [26]. True positives were identified using a linear function of match length and score, using shuffled sequences as negative controls. Nucleotide sequences that did not yield a positive profile hit were discarded.

The remaining nucleotide sequences were clustered at the 97% sequence identity threshold using Uclust [29]. The centroid of each cluster was then retrieved and classified (i) by MLgsc, and (ii) according to the best BLASTX hit against a BLAST database made with the same reference sequences as were used to build the model. In this example, the size of the input dataset was reduced through clustering, but this is not a prerequisite for classification (for example, none of the runs described under “Performance and Comparisons” involve clustering). Since all members of a cluster are assumed to belong to the same OTU, it is therefore only necessary to classify one member of each cluster.

The community composition was determined to genus level, where possible (S1 Table). In the BLAST-based classification, members affiliated to *Paenibacillus* (over 50% of the community), *Bacillus*, *Clostridium*, *Desmospora*, and *Brevibacillus* dominated the community. In comparison, the community composition based on MLgsc was largely dominated by *Clostridium* (73.87%), followed by *Paenibacillus*, and *Bacillus*. Applying an evidence ratio cutoff of 10 (see section Procedure, subsection Validation), 14.05% of the sequences were considered unclassified.

The results of these two classification methods were after compared with a distribution of Firmicutes in the sample, according to a classification of 16S rRNA amplicons. The first obvious difference between the two datasets is the fraction of unclassified sequences, which was more than 60% in the 16S rRNA gene data and 14% in *spo0A*-MLgsc, and only 3% in *spo0A*-BLAST data. Among the identified groups, the genera *Paenibacillus*, *Bacillus*, *Clostridium*, *Geobacillus*, *Alicyclobacillus*, *Anoxybacillus*, *Desulfotomaculum* and *Sporosarcina* were found in the 16S rRNA and *spo0A* gene datasets. For these genera, important differences in their relative abundances were obtained in the RDP-BLAST comparison for all but *Bacillus*. In the RDP-MLgsc comparison, the relative abundances were for the most part consistent, in particular concerning the dominance of *Clostridium*. It is worth mentioning that since the classification with MLgsc was made on protein sequences, a third of the amplicons were filtered out after translation and comparing the ORFs with the Spo0A profile.

## Discussion

Alignment of the query sequence to a PWM takes time proportional to the product of the lengths of the matrix and the sequence. Composition-based methods such as RDP's are typically faster, as k-mers can be counted in time proportional to the length of the sequence only. This disadvantage of MLgsc is partially offset by the use of decision tree: firstly, the sequence is aligned only once (to the matrix at the root of the tree); secondly, in a balanced, bifurcating tree of *n* OTUs, only 2 log_2_(*n*) scorings need to be performed. For the 585 OTUs of the 16S rRNA example, that means only about 20 scorings. The advantage of the divide-and-conquer strategy will be stronger as the number of OTUs grows.

Contrary to phylogeny-based methods like EPA [9], MLgsc does not perform full placement: queries are always assigned to a leaf of the tree (that is, a reference taxon). This has the drawback of potentially “forcing” a query to a taxon of which it is a relative and not a member, but it also avoids the more costly task of estimating the likelihood or posterior probabilities of a tree for each candidate query placement. The use of evidence ratio cutoffs can help determine to which node the classification can be made with confidence.

With default parameters (i.e., no ER cutoff), Mlgsc's accuracy and rate of classification are closest to Mothur with RDP: MLgsc classifies all sequences with an error rate of 2.5%, Mothur-RDP classifies virtually all sequences (99.8%) with an error rate of 3.2%. With an ER cutoff of 20, MLgsc classifies 84% of the queries with an error rate of 0.15%, which approaches the performance of Mothur's KNN method with BLAST search, which classifies 70.54% of the queries with 0.047% error rate. The two other KNN methods of Mothur achieve an error rate of zero, albeit at the cost of failing to classify up to one third of the queries.

In all cases, MLgsc is faster than the Mothur methods. It must be said, however, that all the Mothur methods classify to species level, while for the examples shown here MLgsc was trained to genus level. The Mothur methods may have shorter run times if limited to genus level.

For the NifH protein, both Mlgsc and FrameBot have large error rates. This may be due to the fact that NifH is found in both Archaea and Bacteria, which can conceivably make it more difficult to produce good alignments. In this case, MLgsc was also faster and more accurate than FrameBot.

In an example of application we classified Spo0A sequences from one of our research studies. Classification of environmental sequences is complex, since the true nature of the community composition is unknown. Indeed, large differences were obtained when MLgsc was compared to BLAST. Sequencing and classification of the 16S rRNA were also performed in the same sample. It is clear that the community composition deduced from these two markers (16S rRNA and *spo0A* genes) cannot be compared directly as independent amplification and sequencing biases are another source of inaccuracy. However, it is reassuring that MLgsc produced results that were more compatible with the 16S rRNA gene approach.

Like any classification method, MLgsc is dependent on the quality and comprehensiveness of the references. The fact that many Spo0A sequences yield scores much lower than the references yet higher than negative controls (shuffled sequences, S1 Fig) suggests that much of the diversity of Spo0A has yet to be recovered from the environment. As new reference sequences become available, the proportion of unclassifiable sequences should fall.

## Conclusion

MLgsc is a general, maximum-likelihood sequence classifier that uses phylogenetic information to guide classification. It can classify protein as well as nucleic acid sequences, and is not specialized to any particular taxon, nor to any specific gene or protein. It can achieve accuracy rates comparable to RDP’s with shorter run times. It has a simple, straightforward interface and can be easily integrated in bioinformatics pipelines.

## Supporting Information

S1 Fig Box-plots of MLgsc on Spo0A training and sample sets. The score of classification from original sequences was compared with permutated versions of the same. From left to right, training set (green); shuffled training set (red); environmental sample (blue); shuffled environmental sample (violet).
(TIFF)

S1 Table Comparison of genus distribution derived from 16S rRNA gene and *spo0A* sequence datasets from Lake Geneva. Values are given in sequence counts per genus. The two classification methods used for spo0A assignment (BLAST and MLgsc) are indicated.
(DOCX)
