# Supplementary figures and images for: MLgsc: A Maximum-Likelihood General Sequence Classifier
Source: PLoS One. 2015 Jul 6;10(7):e0129384. doi: 10.1371/journal.pone.0129384 (PMC4492669; doi:10.1371/journal.pone.0129384)

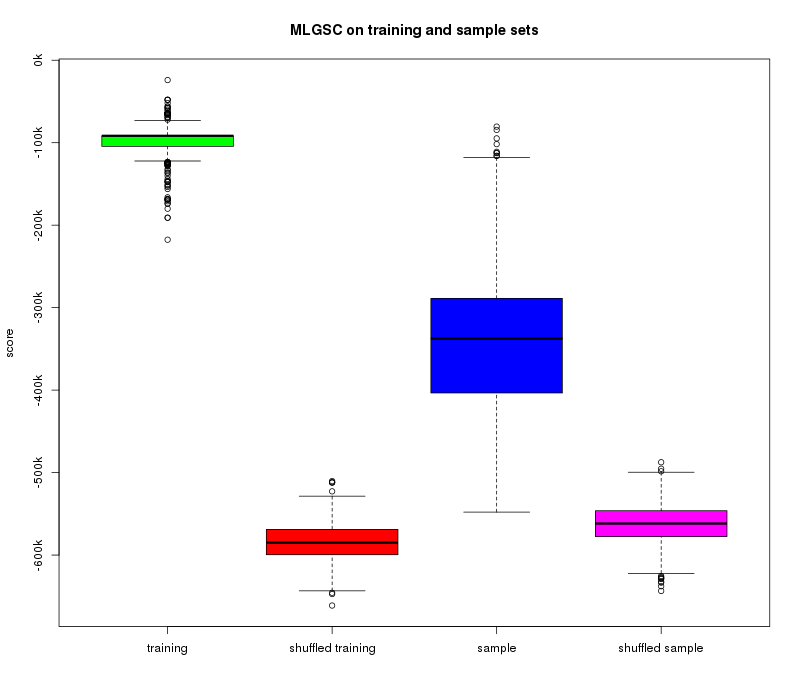

Supplement: S1 Fig — The score of classification from original sequences was compared with permutated versions of the same. From left to right, training set (green); shuffled training set (red); environmental sample (blue); shuffled environmental sample (violet). (TIFF) [file pone.0129384.s001.tiff]
