# Supplementary material for: MLgsc: A Maximum-Likelihood General Sequence Classifier
Source: PLoS One. 2015 Jul 6;10(7):e0129384. doi: 10.1371/journal.pone.0129384 (PMC4492669; doi:10.1371/journal.pone.0129384)
Supplement: S1 Table — Values are given in sequence counts per genus. The two classification methods used for spo0A assignment (BLAST and MLgsc) are indicated. (DOCX) [file pone.0129384.s002.docx]

**Supplementary Table 1.** Comparison of genus distribution derived from 16S rRNA gene and *spo0A* sequence datasets from Lake Geneva. Values are given in sequence counts per genus. The two classification methods used for spo0A assignment (BLAST and MLgsc) are indicated. For MLsgc sequences

|  | Sequence counts (Percentage) | | |
| --- | --- | --- | --- |
|  | 16S rRNA gene | *spo0A* (BLAST) | *spo0A* (MLgsc) |
| *Alicyclobacillus* | 5 (0.05) | 2 (0.21) |  |
| *Anoxybacillus* | 12 (0.11) | 1 (0.11) |  |
| *Bacillus* | 1056 (9.77) | 98 (10.32) | 30 (4.53) |
| *Geobacillus* | 1 (0.01) | 33 (3.37) | 1 (0.15) |
| *Paenibacillus* | 247 (2.29) | 493 (51.89) | 47 (7.10) |
| *Sporosarcina* | 72 (0.67) | 7 (0.74) |  |
| *Clostridium* | 2514 (23.26) | 85 (8.95) | 489 (73.87) |
| *Desulfotomaculum* |  | 10 (1.05) |  |
| *Brevibacillus* |  | 65 (6.84) | 2 (0.30) |
| *Pasteuria* |  | 8 (0.84) |  |
| *Desmospora* |  | 82 (8.63) |  |
| *Lysinibacillus* |  |  |  |
| Not shared | 306 (2.83) | 36 (3.80) |  |
| Not classified to genus | 6596 (61.02) | 30 (3.16) | 93 (14.05) |
